# Hsp90-Dependent Activation of Protein Kinases Is Regulated by Chaperone-Targeted Dephosphorylation of Cdc37

**DOI:** 10.1016/j.molcel.2008.07.021

**Published:** 2008-09-26

**Authors:** Cara K. Vaughan, Mehdi Mollapour, Jennifer R. Smith, Andrew Truman, Bin Hu, Valerie M. Good, Barry Panaretou, Len Neckers, Paul A. Clarke, Paul Workman, Peter W. Piper, Chrisostomos Prodromou, Laurence H. Pearl

**Affiliations:** 1 Section of Structural Biology, Institute of Cancer Research, Chester Beatty Laboratories, 237 Fulham Road, London SW3 6JB, UK; 2 Urologic Oncology Branch, Center for Cancer Research, National Cancer Institute/NIH, 9000 Rockville Pike, Building 10/CRC, Room 1-5940, Bethesda, MD 20892-1107, USA; 3 Cancer Research UK Centre for Cancer Therapeutics, Institute of Cancer Research, Haddow and McElwain Laboratories, Sutton, Surrey SM2 5NG, UK; 4 Johns Hopkins Bloomberg School of Public Health, 615 N. Wolfe Street, Baltimore, MD 21205, USA; 5 Pharmaceutical Science Division, King's College London, Franklin-Wilkins Building, 150 Stamford Street, London SE1 9NN, UK; 6 Department of Molecular Biology and Biotechnology, University of Sheffield, Firth Court, Western Bank, Sheffield S10 2TN, UK

**Keywords:** PROTEINS

## Abstract

Activation of protein kinase clients by the Hsp90 system is mediated by the cochaperone protein Cdc37. Cdc37 requires phosphorylation at Ser13, but little is known about the regulation of this essential posttranslational modification. We show that Ser13 of uncomplexed Cdc37 is phosphorylated in vivo, as well as in binary complex with a kinase (C-K), or in ternary complex with Hsp90 and kinase (H-C-K). Whereas pSer13-Cdc37 in the H-C-K complex is resistant to nonspecific phosphatases, it is efficiently dephosphorylated by the chaperone-targeted protein phosphatase 5 (PP5/Ppt1), which does not affect isolated Cdc37. We show that Cdc37 and PP5/Ppt1 associate in Hsp90 complexes in yeast and in human tumor cells, and that PP5/Ppt1 regulates phosphorylation of Ser13-Cdc37 in vivo, directly affecting activation of protein kinase clients by Hsp90-Cdc37. These data reveal a cyclic regulatory mechanism for Cdc37, in which its constitutive phosphorylation is reversed by targeted dephosphorylation in Hsp90 complexes.

## Introduction

A significant fraction of the protein kinases in a eukaryotic cell require association with the Hsp90-Cdc37 molecular chaperone complex as a prerequisite for activation and/or assembly into functional complexes (reviewed by Pearl [2005]). Indeed, a recent analysis suggests that >75% of the yeast kinome is affected by Cdc37p downregulation or loss of function (Mandal et al., 2007). In mammalian cells where the number of kinases has expanded considerably from the “minimal” yeast set, the proportion of Cdc37-dependent kinases is likely to prove even greater. While Cdc37 in yeast has some ability to stabilize kinases independently of Hsp90 (Lee et al., 2002), a wide range of coprecipitation and inhibition studies show that the majority of Cdc37 clients in mammalian cells are involved with, and dependent on, the Hsp90-Cdc37 chaperone complex (reviewed by Pearl [2005]).

Cdc37 (also known as p50) is a protein kinase-specific adaptor cochaperone, the interaction of which with protein kinases is primarily mediated by the N-terminal segment (Grammatikakis et al., 1999; Shao et al., 2003a), while the middle and C-terminal regions mediate interaction with Hsp90 (Shao et al., 2001; Roe et al., 2004; Zhang et al., 2004). Cdc37 has independent dimerization interfaces in the N-terminal and middle segments of the protein, and associates with Hsp90 in vitro in a dimer-dimer complex in the absence of a bound kinase client (Siligardi et al., 2002; Zhang et al., 2004; Roiniotis et al., 2005). However, studies of in vivo assembled Cdc37 complexes indicate a dynamic oligomerization process in which Cdc37 interacts with kinases as a dimer, but simultaneously with kinase and Hsp90 as a monomer (Vaughan et al., 2006). In addition to its role as an adaptor, and in common with Hsp90 cochaperones, such as Sti1, Aha1, and p23 (Prodromou et al., 1999; Panaretou et al., 2002; Meyer et al., 2004; Ali et al., 2006), Cdc37 has a regulatory function, and can arrest the ATPase-coupled chaperone cycle by preventing ATP-dependent N-terminal association (Siligardi et al., 2002; Roe et al., 2004).

While the basis for its interaction with Hsp90 has been described at the atomic level (Roe et al., 2004), how and where Cdc37 interacts specifically and selectively with protein kinases remains unclear, although there is a general consensus that kinase binding is a function of the N-terminal region of Cdc37 (Pearl and Prodromou, 2006). On the kinase side, a variety of studies indicate interaction with the N-terminal lobe of the kinase client (Scroggins et al., 2003; Prince and Matts, 2004), with the conserved glycine-rich P loop that projects over the ATP binding site also playing an important role (Zhao et al., 2004).

It has become clear that Cdc37 is phosphorylated in vivo at serine 13 (Ser 14/17 in *Saccharomyces cerevisiae*), probably by casein kinase II (CK2), and that the absence of this phosphorylation severely compromises Cdc37 function in vivo (Bandhakavi et al., 2003; Shao et al., 2003b; Miyata and Nishida, 2004). However it is far from clear what function this phosphorylation serves, although its location in the N-terminal region of Cdc37 most obviously implicates it in protein kinase interactions. Nor is it clear whether this phosphorylation is regulated in any way, as CK2 is generally considered to be constitutively active (Litchfield, 2003).

Here we describe studies of Cdc37 phosphorylation in different complexes with protein kinases and with Hsp90, which provide insight into the involvement of pSer13 in Hsp90-Cdc37-kinase (H-C-K) complexes, reveal a clear role for the Hsp90-targeted protein phosphatase PP5/Ppt1 in the chaperone-mediated activation of protein kinases, and suggest a functional mechanism of Cdc37 cycling controlled by chaperone-targeted dephosphorylation.

## Results

### Cdc37-Ser13 Phosphorylation in Cdk4 and Hsp90 Complexes

**Figure 1:**
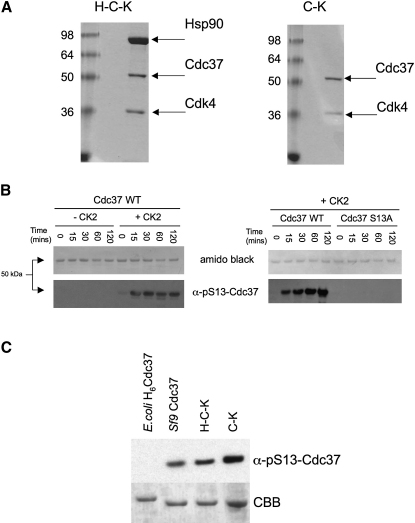
Specificity of the Polyclonal Anti-PhosphoSer13-Cdc37 Antibody (A) Coomasie-stained SDS-PAGE of purified H-C-K and C-K complexes. (B) Incubation of purified WT-Cdc37 expressed in *E. coli* with CK2 at 30°C shows a time-dependent specific signal from the α-pSer13-Cdc37 polyclonal antibody. No signal was observed with an S13A mutant. The blot was amido black-stained as a loading control. It has been shown elsewhere that Ser13 is the only site of phosphorylation by CK2 on Cdc37. (C) Free Cdc37, Cdc37 in complex with Cdk4 (C-K) and Cdc37 in complex with Hsp90 and Cdk4 (H-C-K) expressed in insect cells are all phosphorylated on Ser13.

We used a baculovirus system in *Sf9* cells to express human Cdc37 alone, or with the Cdc37/Hsp90-dependent protein kinase Cdk4. This allowed purification, via an N-terminal His_6_-tag on Cdk4, of two complexes of Cdc37 and Cdk4 (Vaughan et al., 2006)—one devoid of Hsp90, Cdc37-Kinase (C-K), and one which stoichiometrically copurified with the endogenous *Sf9* Hsp90, H-C-K (Figure 1A). *Sf9* Hsp90 is >70% identical to human Hsp90s, and it is safe to assume that it makes authentic interactions with the human proteins, so that the complexes formed are truly representative of endogenous Hsp90-cochaperone-client complexes.

Several studies have highlighted the importance of phosphorylation of Cdc37 on Ser13, in a highly conserved stretch of residues at the N terminus, for recruitment of the cochaperone to Hsp90-client complexes (Shao et al., 2003b), and for activation of those clients (Miyata and Nishida, 2004). To gain further insight into the role and regulation of Cdc37 phosphorylation in formation and stability of complexes with protein kinases and Hsp90, we developed a phosphospecific antiserum for pSer13-Cdc37 as a tool for probing the phosphorylation state of Cdc37 (see Experimental Procedures).

Casein kinase 2 (CK2) has been shown to be the main kinase responsible for phosphorylation of Cdc37 on Ser13 in vivo (Bandhakavi et al., 2003; Shao et al., 2003b; Miyata and Nishida, 2004). To verify the specificity of our antibody, Cdc37 expressed in *Escherichia coli* was incubated with or without CK2 (Promega, UK) and analyzed by Western blot with the α-pSer13-Cdc37 antiserum. The *E. coli*-expressed protein itself was unreactive, but developed a clear signal on incubation with CK2 (Figure 1B), confirming the specific recognition of the phosphorylated Cdc37 by our antibody. Western blots of *Sf9*-expressed Cdc37, and of the C-K and H-C-K complexes, with α-pSer13-Cdc37, showed strong reactive bands, indicating that Cdc37 was substantively phosphorylated on Ser13 in all cases (Figure 1C).

### pSer13 Is Protected against Nonspecific Dephosphorylation in an H-C-K Complex

The requirement of phosphorylation on Ser13 for Cdc37 association with Hsp90-client complexes in vivo, and for activation of those clients, suggests that pSer13 might be involved in mediating Cdc37s interactions with client kinases, and may therefore be buried in a protein-protein interface in the Cdk4 complexes. To probe the accessibility of pSer13-Cdc37, we utilized the phosphospecific antiserum to measure its susceptibility to dephosphorylation in the H-C-K and C-K complexes compared with the isolated, phosphorylated cochaperone.

**Figure 2:**
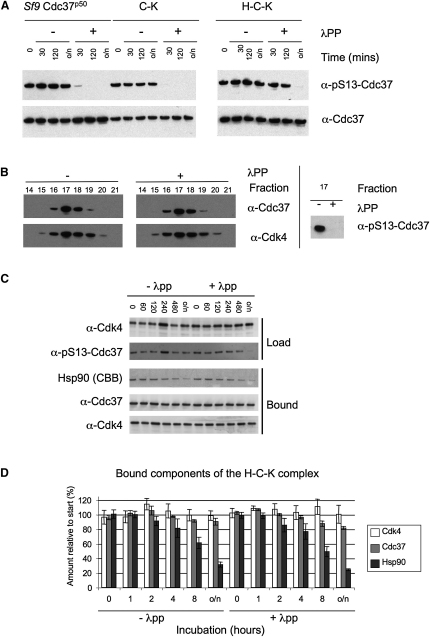
Dephosphorylation of Free Cdc37 or Cdc37 within the C-K and H-C-K Complexes by λPP (A) The rate of dephosphorylation of free Cdc37, the C-K complex, and the H-C-K complex were compared. Samples with approximately equivalent amounts of Cdc37 were incubated in the absence (−) and presence (+) of λPP at room temperature for the specified times. Western blots were probed with α-pSer13-Cdc37. The blots were stripped and reprobed with α-Cdc37 as a loading control. (B) The C-K complex was incubated in the absence (−) and presence (+) of λPP and the effect of dephosphorylation on composition examined by analytical gel filtration with a Superose 6 10/300 GL column; 1 ml fractions were probed for each component of the complex by Western blot. Dephosphorylation was confirmed by probing with α-pSer13-Cdc37. Cdk4 alone elutes in fraction 19 (data not shown). (C) The H-C-K complex was incubated in the absence (−) or presence (+) of λPP. At specified time points, samples were withdrawn and the effect of dephosphorylation on complex stability assessed by pulldown of the complex via the His_6_-tagged Cdk4 on Talon resin. Samples were probed by Western blot for Cdc37 and Cdk4 and Coomassie stain for Hsp90. Dephosphorylation of the coprecipitated samples was confirmed by probing with α-pSer13-Cdc37. (D) The experiment was repeated in triplicate, and the amount of the components precipitated by the Talon resin (Bound) relative to the start of the incubation, was determined by densitometry of the Western blots for Cdk4 and Cdc37, or the Coomassie-stained PAGE gel for Hsp90. Error bars indicate SD of the measurements.

With the isolated Cdc37 and the C-K complex, reactivity to pSer13-Cdc37 antiserum was lost rapidly on incubation with the nonspecific λ-phosphatase (NEB, UK), showing that the N terminus of Cdc37 was fully accessible and could be efficiently dephosphorylated in both cases. In contrast, pSer13 in the H-C-K complex was highly resistant to dephosphorylation, with signal persisting for several hours, suggesting that it is buried or occluded in the ternary complex and inaccessible to the phosphatase (Figure 2A).

Dephosphorylation had no discernable effect on the integrity of the C-K complex, and untreated or dephosphorylated C-K complex behaved identically in analytical gel filtration, with Cdc37 and Cdk4 coeluting in both cases (Figure 2B). Together with its accessibility in the C-K complex, this shows that pSer13 is not essential for maintaining the interaction of Cdc37 and kinase, as previously suggested (Miyata and Nishida, 2004). While the ternary H-C-K complex was resistant, protracted incubation with λ-phosphatase eventually results in loss of pSer13-Cdc37 immunoreactivity (Figure 2B). The H-C-K complex was also unaffected by dephosphorylation, and with both untreated and dephosphorylated complex, precipitation of Cdk4 coprecipitated Cdc37 and Hsp90 (Figure 2C), indicating that it is not essential for integrity of the complex, once assembled. pSer13-Cdc37 may play a subtle conformational role within the H-C-K complex (Shao et al., 2003b), but structural studies will be required to delineate this. However, it plays no essential role in cementing the interaction of Cdc37 or a bound kinase with each other, or of either with Hsp90.

### PP5 Specifically Dephosphorylates Cdc37 in Hsp90 Complexes

As CK2 is considered to be constitutively active (Litchfield, 2003), much of the Cdc37 pool will be phosphorylated on Ser13 much of the time. Although pSer13-Cdc37 appears nonessential for stability of H-C-K complexes in vitro, it is essential for their assembly in vivo, and a Ser13Ala mutation, which prevents phosphorylation, severely compromises recruitment of Cdc37 into Hsp90-kinase complexes (Shao et al., 2003b). More surprising is the observation that mutation of Ser13 to glutamic acid, which often acts as a constitutive phosphoserine mimetic (Potter and Hunter, 1999), also impairs Cdc37 function in vivo. One explanation for this is that it is not just phosphorylation per se, but also its reversibility that is important. Thus, dephosphorylation of Ser13 by a protein phosphatase, as well as its phosphorylation by the protein kinase CK2, may be required for the full biological activity of Cdc37 as a kinase-specific Hsp90 cochaperone in vivo. PP5 (Ppt1p in yeast) is a serine/threonine protein phosphatase recruited to Hsp90 via interaction of its N-terminal TPR domain with the MEEVD motif at the extreme C terminus of the chaperone (Cliff et al., 2005). PP5 has been shown to influence maturation of several Hsp90-dependent clients (Silverstein et al., 1997; Shao et al., 2002; Conde et al., 2005; von Kriegsheim et al., 2006), although there is no consensus regarding the precise nature of its involvement. Deletion of yeast Ppt1p in vivo results in general functional impairment and increased basal phosphorylation of Hsp90, implicating it as one of the targets of Ppt1p (Wandinger et al., 2006). Given its clear association with Hsp90, we sought to determine whether PP5 might be competent to reverse the phosphorylation of pSer13-Cdc37.

**Figure 3:**
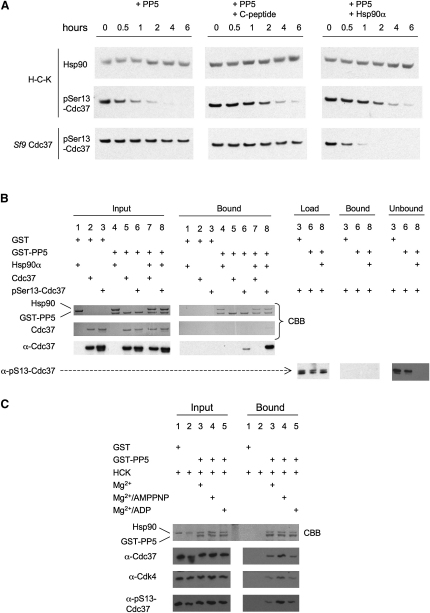
Dephosphorylation of Cdc37 by PP5 In Vitro (A) H-C-K complex or *Sf9*-expressed Cdc37 was incubated with PP5 at room temperature and the extent of dephosphorylation at specified times assessed by probing Western blots with α-pSer13-Cdc37. This experiment was repeated in the presence of a 10-fold molar excess of PP5 activator—either a seven residue peptide (SRMEEVD) corresponding to the TPR binding motif of Hsp90 or full-length human Hsp90α. (B) The interaction of PP5 with Cdc37 is mediated by Hsp90. GST-tagged PP5 was incubated with Cdc37 or phosphorylated Cdc37 in the presence and absence of Hsp90 at 4°C. A small fraction of pSer13-Cdc37 is found in complex with GST-PP5, but the majority of Cdc37 remains phosphorylated; this interaction is significantly increased in the presence of Hsp90, and coincides with an increase in PP5 activity toward Cdc37 (Unbound, lanes 6 and 8). The affinity of GST-tagged PP5 for Hsp90-bound Cdc37 is low compared with that for Hsp90-bound pSer13-Cdc37 (10% Bound, lanes 7 & 8). CBB = Coomassie Brilliant Blue. (C) The interaction of PP5 with the H-C-K complex. GST-tagged PP5 was incubated with the H-C-K complex at 4°C, and could coprecipitate all components of the complex. The interaction was stabilized, and the activity of PP5 reduced, by inclusion of AMPPNP, but not ADP.

In contrast to our observation with the nonspecific λ-phosphatase, pSer13-Cdc37 in isolation was almost completely resistant to dephosphorylation by PP5. However, when PP5 was added to the purified H-C-K complex, again in contrast to λ-phosphatase, we observed efficient dephosphorylation of the pSer-Cdc37 in the complex, suggesting specific targeting of PP5 to a pSer13-Cdc37 substrate by the Hsp90-kinase complex (Figure 3A, left panel).

The basal activity of PP5 is low due to autoinhibition by its TPR domain. Binding of the TPR domain to the C-terminal MEEVD peptide of Hsp90 promotes a conformational change, relieving autoinhibition and activating the phosphatase ∼7-fold (Yang et al., 2005). Thus, the activity toward pSer13-Cdc37 in the H-C-K complex might not be due to specific targeting by Hsp90, but activation of PP5 *in trans* by Hsp90 present in the reaction. In order to test this, we incubated pSer13-Cdc37 and the H-C-K complex with PP5 as before, but with a 10-fold molar excess of an Hsp90 C-terminal peptide, which, as previously shown (Yang et al., 2005), binds to the TPR domain and activates the phosphatase. Despite explicit activation of PP5 by the Hsp90 peptide, isolated pSer13-Cdc37 remained completely resistant, while dephosphorylation of the H-C-K complex was actually reduced, due to competitive blockade of the PP5 TPR domain required for targeting to Hsp90 by the Hsp90 peptide (Figure 3A, middle panel). Finally, we looked at the effect of full-length Hsp90α on dephosphorylation of the two Cdc37 substrates. As with the Hsp90 peptide, addition of free Hsp90α, which activates PP5, reduced H-C-K dephosphorylation due to competition for the PP5-TPR domain. However, the presence of full-length Hsp90α, the N-terminal domain of which provides the binding site for Cdc37 (Roe et al., 2004), enabled rapid dephosphorylation of the isolated pSer13-Cdc37, which was otherwise highly resistant to PP5 (Figure 3A, right panel).

For Hsp90-dependent dephosphorylation to occur *in cis* implies that Cdc37 and PP5 must bind simultaneously to the same Hsp90 dimer. In confirmation of this, coprecipitation of pSer13-Cdc37 by GST-PP5, increased substantially when Hsp90 was present, indicating an Hsp90-bridged interaction between the two cochaperones (Figure 3B). Consistent with this, dephosphorylation in the absence of Hsp90 was negligible (Figure 3B, Unbound). The relative Hsp90-mediated affinity of PP5 for pSer13-Cdc37 was higher than for nonphosphorylated Cdc37 (Figure 3B, compare lanes 7 and 8), confirming that pSer13-Cdc37 is a bona fide substrate for PP5. Significantly, GST-PP5 also coprecipitated Cdc37 and Cdk4 when incubated with the in vivo assembled H-C-K complex (Figure 3C).

Nucleotides affected the degree of dephosphorylation achieved by PP5, with the complex resistant to dephosphorylation by PP5 in the presence of the nonhydrolyzable ATP analog AMPPNP, but not with ADP or ATP. This suggests that the conformation of Hsp90 induced by AMPPNP further stabilizes a buried binding site for pSer13-Cdc37 within the H-C-K complex.

Taken together, these in vitro data show that PP5 can efficiently dephosphorylate Cdc37 only in the specific context of an Hsp90 complex that provides binding sites for both cochaperones simultaneously. Furthermore, binding of PP5 to an H-C-K complex activates the phosphatase function of PP5, and may conformationally remodel the H-C-K complex to expose the N-terminal pSer13 site on Cdc37, which is otherwise occluded and relatively resistant to dephosphorylation by a nonspecific phosphatase.

### PP5/Ppt1p Interacts with and Dephosphorylates Cdc37 In Vivo

*S. cerevisiae* has orthologs for Hsp90, PP5, and Cdc37, and phosphorylation on Ser14/Ser17 by yeast CK2 is essential for yeast Cdc37p function in vivo (Bandhakavi et al., 2003). Yeast, therefore, provides a genetically tractable model for testing in vivo the significance of Hsp90-targeted dephosphorylation of pCdc37 by PP5 demonstrated in vitro with human proteins.

**Figure 4:**
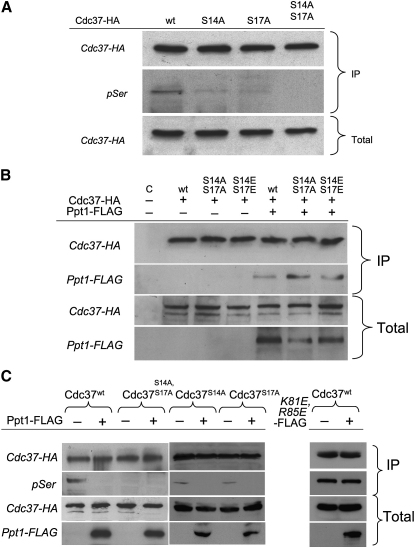
Dephosphorylation of Cdc37p by Ppt1p in Yeast Extracts (A) Cdc37p is phosphorylated on both S14 and S17 in yeast cell extracts. Immunoprecipitated (IP) Cdc37-HA and its S14A and S17A mutants were immunoblotted with anti-Phospho-Serine antibody. (B) FLAG-tagged Ppt1p coimmunoprecipitates with the wild-type (WT), nonphosphorylatable (S14A/S17A), and phosphomimic (S14E/S17E) forms of HA-tagged Cdc37p. (C) HA-tagged wild-type Cdc37p immunoprecipitated from cells overexpressing FLAG-tagged Ppt1p is substantially dephosphorylated, as are the single S14A and S17A phosphorylation-site mutants. Overexpression of Ppt1 has no effect on the phosphorylation of HA-tagged Cdc37p in cells expressing the nonphosphorylatable S14A/S17A mutant. A FLAG-tagged Ppt1p K81E/R85E mutant, which abrogates Ppt1 interaction with Hsp90, no longer dephosphorylates HA-tagged Cdc37p.

We expressed an HA-tagged Cdc37p in yeast and showed that phosphoserine could be detected in the immunoprecipitated protein with a α-pSer monoclonal antibody (QIAGEN) (Figure 4A). We observed weak phosphoserine reactivity in HA-immunoprecipitated Cdc37p with single S14A and S17A mutations, but none when both serines were mutated, consistent with observations that both residues are phosphorylated by CK2 in yeast (Bandhakavi et al., 2003). We then additionally overexpressed FLAG-tagged Ppt1p and used an immunoprecipitation assay to determine whether both cochaperones could occur in the same Hsp90 complex in vivo. FLAG-tagged Ppt1p could be readily immunoprecipitated by wild-type HA-tagged Cdc37p, and by the phosphorylation-resistant S14A/S17A mutant, or phosphomimetic S14E/S17E mutant (Figure 4B). Significantly, no phosphoserine was detected in wild-type HA-Cdc37p immunoprecipitated from cells overexpressing wild-type Ppt1p. However, this signal was not lost on overexpression of a Ppt1 mutant with mutations (K81E/R85E), known to abrogate TPR domain interaction with the C terminus of Hsp90 (Russell et al., 1999; Scheufler et al., 2000). The failure of the Ppt1 K81E/R85E to dephosphorylate Cdc37p in vivo confirms the in vitro observation that PP5/Ppt1 is specifically targeted to Cdc37 by their simultaneous interaction with Hsp90 (Figure 4C).

### Functional Interplay of Cdc37 and PP5/Ppt1 In Vivo

We have shown that PP5/Ppt1p can dephosphorylate Cdc37 in vitro and in vivo in yeast, suggesting a direct role in regulating Cdc37 phosphorylation. As phosphorylation of Cdc37 is essential to its function in mammalian and yeast systems, we sought to determine the consequences of such a regulatory role for PP5/Ppt1p with the yeast system.

**Figure 5:**
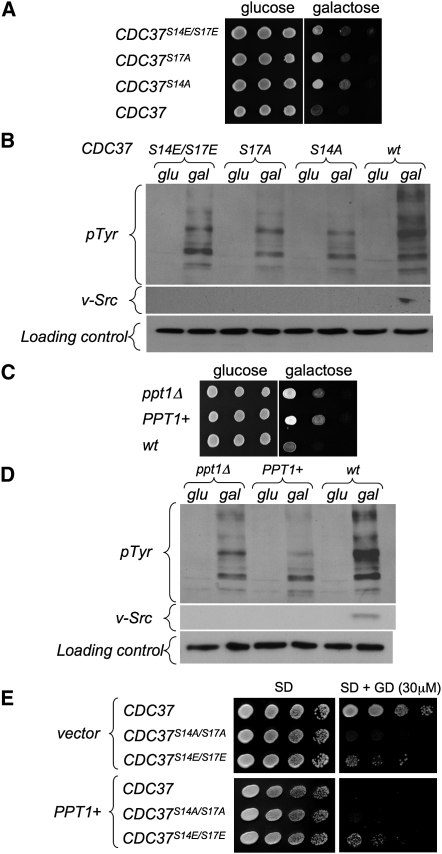
Effects of Cdc37 Mutation and Overexpression/Deletion of Ppt1 on v-Src Expression in Yeast (A) Growth of wild-type (CDC37) cells and strains with S14A, S17A, or the doubly mutated S14E/S17E Cdc37 containing the toxic vSrc gene controlled by the GAL promoter, on glucose (glu)- or galactose (gal)-containing medium. Both nonphosphorylatable and phosphomimetic mutants of Cdc37 compromise vSrc activation. (B) The same strains analyzed for total pY and v-Src levels. Appreciable levels of v-Src were only detectable in wild-type cells. (C) Both the loss (*ppt1Δ*) and overexpression (*PPT1^+^*) of Ppt1p compromise v-Src expression in cells containing the toxic v-Src gene controlled by a GAL promoter. (D) The same strains analyzed for total pY and v-Src levels. As for Cdc37 phosphorylation mutants, appreciable levels of v-Src were only detectable in wild-type cells. (E) Overexpression of Ppt1p causes GA sensitivity in yeast. Cells expressing the wild-type (CDC37), nonphosphorylatable (S14A/S17A), or phosphomimic (S14E/S17E) Cdc37p were transformed with either empty vector or the PPT1 overexpression plasmid (MET25-PPT1). A 1:10 dilution series was grown (4 days, 30°C) on SD agar lacking methionine, without or with 30 μM GA. Overexpression of Ppt1 renders cells as sensitive as the nonphosphorylatable S14A, S17A mutant, of Cdc37.

The tyrosine kinase v-Src is a well-known Hsp90-Cdc37-dependent client (Brugge, 1986; Xu and Lindquist, 1993), the activity of which in yeast is a widely used reporter for analysis of functionality in the Hsp90 chaperone system (Nathan and Lindquist, 1995; Panaretou et al., 2002). Through the use of an inducible expression system (Panaretou et al., 2002), we assessed the effect of mutations in the Cdc37p phosphorylation sites on expression of active v-Src. As v-Src is toxic to yeast, which possesses few bona fide tyrosine kinases, expression of active v-Src can be monitored by cell viability and total phosphotyrosine (pY) incorporation. Compared to wild-type, where v-Src induction was lethal, S14A and S17A phosphorylation-resistant mutants, or the double S14E/S17E constitutive phosphomimetic mutant, rendered cells partially resistant to killing by v-Src (Figure 5A). Consistent with this, these mutants showed decreased pY signals in cell lysates after induction, and v-Src protein was only detectable in wild-type lysates (Figure 5B). The failure of both classes of Cdc37 mutant to fully support v-Src expression directly implicates regulated posttranslational modification at these sites, in client kinase activation.

As Ppt1p overexpression substantially decreased phosphorylation of Cdc37p in Hsp90 complexes in yeast in vivo, and Cdc37p mutations that prevent or constitutively mimic phosphorylation disrupt v-Src activation in vivo, we looked for a direct effect of Ppt1p on v-Src activation. Consistent with earlier observations in mammalian cells with a phosphatase inhibitor (Mimnaugh et al., 1995), v-Src activation was substantially reduced in yeast where Ppt1p was deleted, in a parallel with the effect of Cdc37 phosphorylation-mimetic mutations (Figures 5C and 5D). However, v-Src activation was also significantly impaired in cells overexpressing Ppt1p, as observed for nonphosphorylatable Cdc37 mutants, confirming a regulatory role for Ppt1p in kinase activation in vivo, and suggesting a direct mechanistic effect of the chaperone-targeted dephosphorylation of Cdc37p by Ppt1p.

These data show a clear functional interaction of Cdc37p and Ppt1p in the activation of an exogenous client protein. In order to analyze the functional interplay of Cdc37p and Ppt1p in the Hsp90 system in general, we took advantage of the observation that yeast often remains viable after mutation or downregulation of many Hsp90 cochaperones, but display phenotypes when the functionality of the chaperone system is impaired by Hsp90 inhibitors, such as radicicol or geldanamycin (GA) (Piper et al., 2003). Thus, while GA is only slightly toxic to cells with wild-type Cdc37, it is lethal to cells expressing the double phosphorylation-resistant S14A/S17A mutant (Figure 5E). The S14E/S17E mutant, which mimics phosphorylated Cdc37, retains some resistance to GA, but is far more sensitive than the wild-type. Overexpression of Ppt1p, which severely reduces the level of Cdc37 phosphorylation in Hsp90 complexes, has a strong synthetic lethal effect with GA, making wild-type Cdc37 cells as sensitive as the S14A/S17A mutant. While this could be a consequence of Ppt1p activity on Hsp90 itself, the GA sensitivity of the S14E/S17E mutant was unaffected by Ppt1p overexpression, confirming that the synthetic lethality is a result of Ppt1p activity toward Cdc37p phosphorylated at these sites, and is consistent with their irreversible phosphorylation mimicry.

**Figure 6:**
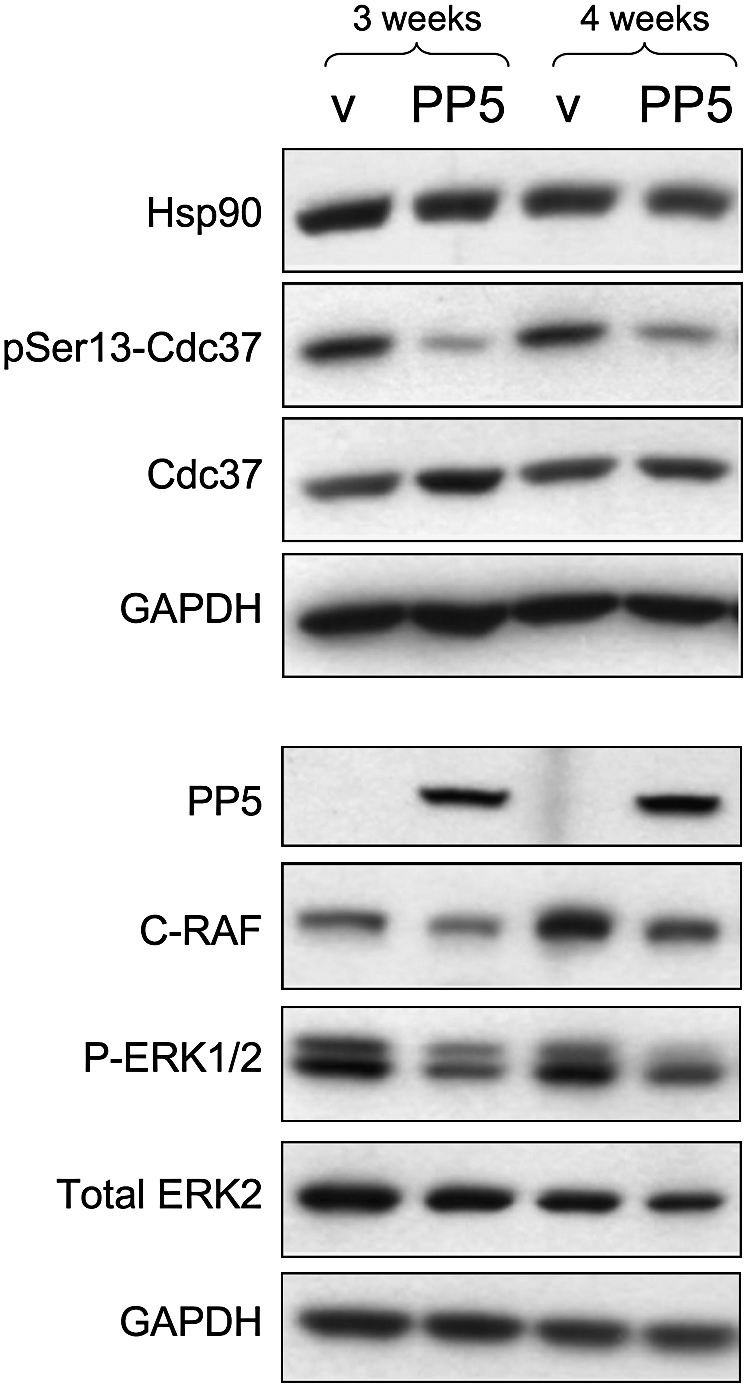
PP5 Dephosphorylates Cdc37 in Tumor Cells and Compromises C-Raf Function Cell lysate of HCT116 cells stably transfected with empty vector (v) or a PP5 expression plasmid (PP5). After 3 weeks, decreased levels of total C-Raf and activated ERK (phospho-ERK1/2) were observed. The effect was amplified further after an additional week of PP5 overexpression.

Finally, we examined the effect of PP5 overexpression on Cdc37 phosphorylation in a human tumor cell line. As in yeast, transfected HCT116 human colon carcinoma cells overexpressing PP5 showed a marked decrease in pSer13-Cdc37 levels detected in cell lysates compared with vehicle-transfected cells, but with no decrease in total Cdc37 (Figure 6). Consistent with the drop in pSer13-Cdc37 levels, we observed a consistent decrease in total levels of C-Raf/Raf1, a known Hsp90-Cdc37-dependent client kinase in the overexpressing cells. We also observed a clear decrease in detectable levels of the activated phosphorylated form of ERK (but not total ERK), which is not an Hsp90 client protein, but a downstream target of C-Raf/Raf1. Taken together, these data show a clear effect of PP5 on pSer13-Cdc37, with diminished Cdc37 phosphorylation, accompanied by a decrease in signaling through the Hsp90-dependent MAP kinase cascade.

## Discussion

The N terminus of Cdc37 has consistently been shown to be required for kinase binding (Grammatikakis et al., 1999; Shao et al., 2001; Ren et al., 2007), while its C terminus is responsible for interaction with Hsp90 (Shao et al., 2001; Roe et al., 2004; Zhang et al., 2004). Nonetheless, the extreme N terminus also plays a role in Hsp90 interactions in the context of a client protein complex. Thus, while mutation of residues 2–4 of Cdc37 impairs binding of client kinase but not of Hsp90 (Shao et al., 2003a), mutation of Trp 7 compromises both. Within the N terminus, phosphorylation of Ser13 (Ser14/17 in yeast) is essential for formation of productive Hsp90-Cdc37-client complexes in vivo (Bandhakavi et al., 2003; Shao et al., 2003b; Miyata and Nishida, 2004).

Through the use of a phosphospecific antiserum to pSer13-Cdc37, we find this phosphorylation present in free Cdc37, and in Cdc37-Cdk4 and Hsp90-Cdc37-Cdk4 complexes, expressed in insect cells (Vaughan et al., 2006). Independently, with a different antibody, Ser13 phosphorylation has been observed in Hsp90-Cdc37 complexes with Cdk4, MOK, v-Src, and C-Raf/Raf1 purified from mammalian cells (Miyata and Nishida, 2007). The presence of pSer13-Cdc37 in complexes with such a diverse set of protein kinases suggests that this is the form in which Cdc37 generally exists in Hsp90 complexes with unactivated kinase clients.

The kinase responsible for phosphorylation of Ser13 is CK2 (Miyata and Nishida, 2004), a Ser/Thr kinase with over 300 diverse substrates, which is considered to be constitutively active (Litchfield, 2003). In some systems, CK2 activity may be regulated by targeting subunits (Pinna, 2002). However, purified *E. coli*-expressed Cdc37 is phosphorylated in vitro, indicating that, as with most CK2 substrates, no “scaffold” factors are required, and specificity is dictated by the peptide sequence alone. Consistent with this, we find that, in addition to Cdc37 purified as part of kinase and Hsp90 complexes, free Cdc37 overexpressed in *Sf9* cells is phosphorylated on Ser13, suggesting that CK2 is constitutively active toward this substrate in vivo.

**Figure 7:**
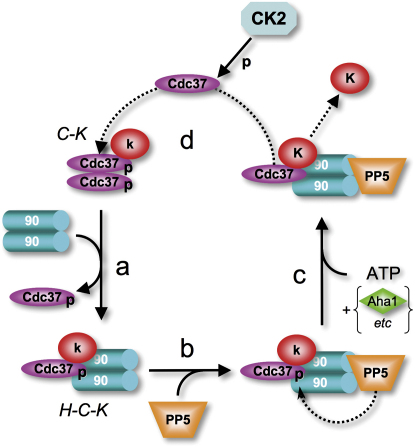
Functional Cycle of Cdc37 Driven by Constitutive Phosphorylation and Targeted Dephosphorylation Schematic of coupling of CK2 phosphorylation and Hsp90-targeted dephosphorylation by PP5/Ppt1 to the role of Cdc37 in assembly and Hsp90-dependent activation of kinase clients. A C-K complex containing an inactive kinase binds an Hsp90 dimer with loss of one Cdc37 to give a stable H-C-K complex, as previously described (Vaughan et al., 2006) (a). Cdc37 is phosphorylated in both these complexes (see above). PP5/Ppt1 can bind to Hsp90 in the H-C-K complex (b) and dephosphorylate Cdc37 prior to, or concomitant with, binding of ATP and other cochaperones, such as Aha1 (e.g., [c]), generating an activated kinase and dissociating the Hsp90-based complex. Free Cdc37 is rephosphorylated by the constitutive activity of CK2 (d) to regenerate the active pSer13 form of the cochaperone, poised for reengagement in a new round of Hsp90-dependent kinase activation.

While phosphorylation of Ser13 by CK2 may be constitutive, we have provided compelling evidence that its reversal is a regulated phenomenon, equally important for Cdc37's biological function. This is in accord with an earlier proposal that it is removal of CK2 phosphorylations, rather than their addition, that is the regulated process (Pinna, 1990). We have demonstrated unequivocally that the Hsp90-targeted protein phosphatase PP5/Ppt1 can dephosphorylate pSer13-Cdc37 only when both cochaperones are bound to the same Hsp90 dimer. Furthermore, we have shown that PP5/Ppt1 and Cdc37 simultaneously associate with Hsp90 complexes in vitro and in vivo, and that PP5/Ppt1 dephosphorylates Cdc37 in such complexes. We are unable to account for the observation that yeast Ppt1 in vitro did not dephosphorylate yeast Cdc37 phosphorylated in vitro by CK2, either in the absence or presence of Hsp90 (Wandinger et al., 2006). However, we note that the protein concentrations used in that study were lower than the known dissociation constants for their interaction. PP5/Ppt1 overexpression reduces pSer13-Cdc37 levels in a human tumor line and impairs activation of kinase clients, such as v-Src or C-Raf, in yeast and human cells. Thus, PP5/Ppt1 acts antagonistically to CK2, generating a compartmentally regulated process in which Cdc37 is phosphorylated, allowing it to recruit a kinase to Hsp90, where PP5/Ppt1 specifically dephosphorylates it. Phosphorylation and dephosphorylation thus drive a directional cycle of Cdc37 function in kinase activation (Figure 7).

Cdc37 dephosphorylation in vitro does not affect the stability of in vivo-assembled complexes, suggesting that dephosphorylation does not result in major changes in affinity between the components of the H-C-K complex. It may, however, cause more subtle conformational changes that affect subsequent activation and release of kinase in the cellular context. Accessibility of pSer13-Cdc37 to PP5 itself appears to be governed by the conformation of the H-C-K complex, and requires the flexibility of the ADP-bound state, rather than the rigidity of the AMPPNP-stabilized, closed conformation (Ali et al., 2006). Dephosphorylation of Cdc37 and the ATPase-coupled client protein activation process may thus be directly coupled. Alternatively, the phosphorylation status of Cdc37 may not alter the affinity of the components of the H-C-K complex for each other, but might alter their affinity for other cochaperones (e.g., Hop, Hsp70/40) that may be involved in later stages of Hsp90-dependent kinase activation, or of the kinase itself for a downstream partner, such as a cyclin or activating kinase.

## Experimental Procedures

### Expression and Purification of Protein

Expression of Cdc37 (Siligardi et al., 2002), human Hsp90α (Millson et al., 2007) and the Hsp90-Cdc37-Cdk4 (H-C-K) and Cdc37-Cdk4 (C-K) complexes (Vaughan et al., 2006) have been previously described. The Cdc37 S13A mutant was constructed with the QuikChange Site-Directed Mutagenesis kit (Stratagene), and the protein was expressed and purified as described for wild-type. *Sf9* expressed Cdc37 was obtained as a by-product of the H-C-K complex purification. GST-PP5 was expressed in B834 cells and purified by glutathione Sepharose and gel filtration on Superdex 200 HR26/60 (GE Healthcare). PP5 protein (residues 16–499) was a generous gift of Jing Yang.

### Antibodies

Polyclonal antibodies (Eurogentec Ltd.) were raised to a phosphopeptide corresponding to residues 9–19 of human Cdc37. Other antibodies were: polyclonal α-Cdk4 (H303) (Santa Cruz Biotechnology); monoclonal α-Cdc37 (C1) (Affinity BioReagents); PP5 (C-20), Cdc37 (H-271), C-Raf (C-20) (Santa Cruz Biotechnology); P-ERK1/2 (no. 9101, Cell Signaling); total ERK2 (a gift of Chris Marshal, ICR); and GAPDH (Chemicon). Secondary antibodies were horseradish peroxidase-linked anti-mouse and anti-rabbit IgG (GE Healthcare) and anti-goat (Abcam).

### Testing Specificity of α-pSer13-Cdc37 Antibody

Cdc37, wild-type, and S13A mutant (*E. coli* expressed), were incubated at 30°C with 0.1 mM ATP, 1 mM MgCl_2_ in 25 mM Tris, and 100 mM NaCl (pH 7.5) in the absence or presence of CK2 (Promega). At each time point, samples were processed as for dephosphorylation reactions. For preparative phosphorylation, Cdc37 was further purified with a Resource Q resin (GE Healthcare) after incubation with CK2 in order to ensure a homogeneously phosphorylated sample.

### Dephosphorylation Reactions

Phosphatase reactions (in 50 mM Tris, 1 mM EDTA, 5 mM DTT, 2 mM MnCl_2_ [pH 7.5]) were stopped by boiling in SDS loading buffer for 5 min, and analyzed by SDS-PAGE and Western blot.

For dephosphorylation by λPP, Cdc37, C-K, and H-C-K with approximately equal concentrations of Cdc37 were incubated with λPP at a 1/5 (w/w) λPP:Cdc37 ratio at room temperature with a final concentration of 2 mM Na_2_MoO_4_. At specified time points, aliquots were removed and processed as described above. For gel filtration, C-K samples were incubated with/without λPP (1:10 ratio) for 1 hr at room temperature and loaded on a Superose 6 HR10/30 column (GE Healthcare) in 50 mM Tris (pH 7.5), 10 mM KCl, 10 mM MgCl_2_, 150 mM NaCl, 1 mM EDTA, 3 mM DTT. For H-C-K dephosphorylation and pulldowns, samples were incubated with/without λPP (10:1 ratio) at 4°C. H-C-K was buffer exchanged into 50 mM Tris (pH 7.5), 10 mM KCl, 10 mM MgCl2, and 150 mM NaCl immediately prior to setup. Phosphatase reaction buffer was modified to exclude DTT and EDTA and to include 150 mM NaCl. At specified time points, aliquots were removed and samples incubated with BSA-blocked Talon resin at 4°C for 15 min. Resin was washed with pulldown buffer (50 mM Tris [pH 7.5], 10 mM KCl, 10 mM MgCl_2_, 150 mM NaCl, 20 mM Na_2_MoO_4_, 0.1% NP40) and bound samples eluted by boiling with loading buffer. Proteins were quantitated by immunochemifluorescence for Cdc37 and Cdk4 (ECL plus; GE Healthcare); Coomassie staining was used for Hsp90. Data were analyzed with ImageQuant (GE Healthcare). In order to confirm H-C-K bound specifically to the resin, the experiment was repeated with a sample of H-C-K preincubated with PreScission protease (GE Healthcare) to remove the His_6_ tag (data not shown).

For dephosphorylation with PP5, Cdc37, C-K, and H-C-K with a [Cdc37] ∼800 nM were incubated at room temperature with 35 nM PP5 and 10-fold excess of human Hsp90α, or with a C-terminal Hsp90 peptide (SRMEEVD). PP5 and activator were preincubated together for 1 hr and sample buffer exchanged into 25 mM Tris and 100 mM NaCl (pH 7.5) immediately prior to setup. Activation of PP5 by Hsp90α or the peptide was confirmed with the colorimetric substrate pNPP, as previously described (Yang et al., 2005) (data not shown).

### Coprecipitation of PP5 with Cdc37

Equal amounts of *E. coli*-expressed Cdc37, either unphosphorylated or phosphorylated in vitro with CK2, and preincubated with/without Hsp90α for 1 hr, were incubated with GST-PP5 or GST at 4°C for 2 hr with glutathione Sepharose resin (GE Healthcare), preequilibrated with pulldown buffer plus BSA (50 mM HEPES [pH 7.5], 150 mM NaCl, 0.1% NP40). Unbound protein was collected, the resin washed, and bound protein eluted by boiling in SDS-PAGE loading buffer. H-C-K was buffer exchanged into pulldown buffer without NP40 immediately prior to incubation with GST or GST-PP5, with or without Mg^2+^ (5 mM) and nucleotide (1 mM), and samples incubated overnight with glutathione Sepharose resin.

### Overexpression of PP5 in Human Cancer Cells

The human colon carcinoma cell line HCT116 was cultured at 37°C, 5% CO_2_ in Dulbecco's modified Eagle's medium (Sigma), supplemented with MEM NEAA, 2 mM glutamine (GIBCO), and 10% FCS (PAA). PP5 open reading frame was amplified from HT29 total RNA by RT-PCR and cloned into the bicistronic eukaryotic expression vector pEFIRES-P (Hobbs et al., 1998). HCT116 cells were transfected with Lipofectamine 2000 (Invitrogen), and cells overexpressing PP5 or the empty vector were selected by culturing with 3 μg/ml puromycin.

### Immunoblotting

Cells were harvested with trypsin/EDTA and washed once in PBS. Protein samples were prepared in lysis buffer (Cell Signaling) with complete mini protease inhibitor cocktail (Roche) and quantitated by BCA protein assay (Pierce). Equal amounts of protein (25 μg) were separated by electrophoresis through 4%–20% Tris-glycine gels (Invitrogen) and transferred onto nitrocellulose. Blocking and primary antibody incubations were in casein buffer (0.5% casein, 10 mM Tris base, 150 mM NaCl, and 0.5 mM thimerosal). Specific antigen-antibody interaction was detected with a horseradish peroxidase-conjugated secondary IgG by chemiluminescence (SuperSignal West Pico chemiluminescent substrate; Pierce) and autoradiography.

### Yeast Strains and Plasmids

*PPT1* plus 500 bp upstream was cloned into pRS314 as EcoRI/XhoI for expression under its native promoter. *PPT1* was placed under *MET25* promoter control in pUT36 SpeI/XhoI. In both constructs, Ppt1 was FLAG tagged at the C terminus. K81E/R85E mutations were introduced with QuickChange site-directed mutagenesis kit (Stratagene) and verified by DNA sequencing.

The yeast strain (BY4741, *ppt1Δ kanMX4*) was from EUROSCARF. The Cdc37(WT), *cdc37*^S14A^, *cdc37*^S17A^, *cdc37*^S14/17A^, and *cdc37*^S14/17E^ strains were generously provided by M. Siderius and S.M. van der Vies (Hawle et al., 2007).

### Growth Conditions

Yeast was grown on YPD (2% [w/v] Bacto peptone, 1% yeast extract, 2% glucose or (YPGal) 2% galactose, 20 mg/L adenine). Selective growth was on dropout 2% glucose (DO) medium supplemented with appropriate amino acids. For agar growth GA sensitivity assays, overnight DO cultures (without methionine) were diluted to an optical density of 600 nm of 0.5, and ≈5 μl aliquots of a 10-fold dilution series were spotted onto DO-2% agar (without methionine) plates supplemented with the indicated level of GA. Growth was monitored over 3–5 days at 30°C.

### v-Src Activation Assay In Vivo

*ppt1Δ* and various *cdc37* mutant alleles with or without overexpressing *PPT1* were transformed with the YpRS316v-SRC (Nathan and Lindquist, 1995; Panaretou et al., 2002). v-SRC is under control of the *GAL1* promoter. Its induction and activation was analyzed as described previously (Panaretou et al., 2002), with the exception that yeast cells were grown onto 2% glucose in order to repress *GAL1* promoter. Cells were also spotted onto YPD or YPGal-2% agar. v-Src protein levels were detected with EC10 mouse antibody (Millipore) and v-Src activity with 4G10 mouse anti-pY antibody (Millipore).
